# Treatment of a *STAT5b::RARα* positive case of APL in a patient not eligible for intensive chemotherapy

**DOI:** 10.1007/s11845-024-03751-0

**Published:** 2024-07-20

**Authors:** Jason Patterson, Kathryn Clarke, Katya Mokretar, Manisha Maurya, Amy Logan, Nicholas Cunningham, Mark Catherwood, Mary Frances McMullin

**Affiliations:** 1 Department of Haematology, Belfast City Hospital, Belfast, Northern Ireland UK; 2 Regional Molecular Diagnostics Service, Belfast City Hospital, Belfast, Northern Ireland UK; 3 Centre for Medical Education, Queen’s University Belfast, Belfast, Northern Ireland UK; 4 Cancer Genetics, Guy’s Hospital, Synnovis, London, UK; 5 Precision Medicine Centre, Queen’s University Belfast, Belfast, Northern Ireland UK

**Keywords:** Acute promyelocytic leukaemia, Low-dose cytarabine, Next generation sequencing, Variant, Venetoclax

## Abstract

Acute promyelocytic leukaemia (APL) with a *STAT5b::RARα* gene fusion is an extremely rare subtype of APL characterised by resistance to conventional therapies and extremely poor prognosis. This case highlights that whilst APL with variant RARα translocations are rare, they do pose significant challenges both diagnostically and in their clinical management. This case, in the first instance, demonstrates the importance of using a combination of molecular techniques including next generation sequencing (NGS) for diagnosis particularly in morphological and immunophenotypic typical APL which appears negative by confirmatory testing. Secondly, our patient represents, to the best of our knowledge, the first documented example of this rare disease that has been managed with, and shown sensitivity to low-dose cytarabine (LDAC) in combination with venetoclax (Ven). This case demonstrates that although treatment options are extremely limited for patients not eligible for intensive chemotherapy non-intensive options do show increasing promise.

## Introduction

Acute promyelocytic leukaemia (APL) is a distinct sub-class of acute myeloid leukaemia (AML) as described by the most recent 2022 World Health Organisations (WHO) classification of haematological malignancies [1]. The disease is most commonly cytogenetically characterised by a unique chromosomal translocation involving the retinoic acid receptor alpha (*RARα*) gene on chromosome 17 and the promyelocytic leukaemia (*PML*) gene on chromosome 15, resulting in the formation of the *PML::RARα* gene fusion [2]. This understanding of the pathogenesis of APL has led to the introduction of highly effective targeted therapies such as all-trans retinoic acid (ATRA) and arsenic trioxide (ATO) [3]. The use of these agents has been shown to be remarkably successful when treating non-high-risk APL patients with an event free survival after 72 months of 96.6% having been achieved [4].

The *PML::RARα* fusion gene is detectable in approximately 98% of patients with APL using fluorescence in situ hybridisation (FISH) or reverse transcription polymerase chain reaction (RT-PCR). However, in the remaining 1–2% of cases of morphologically typical APL novel, extremely rare fusion proteins have been identified involving *RARα* and cryptic gene partners excluding *PML*. These are often clustered according to their sensitivity to ATRA therapy. Those that are sensitive include *NPM1::RARα* and *NuMa::RARα* and those that are resistant include the fusion genes; *PLZF::RARα, ZBTB16::RARα* and *STAT5b::RARα* [5]. The latter are also frequently resistant to ATO and standard induction chemotherapy regimens and are therefore associated with much worse outcomes [6].

The APL variant characterised by *STAT5b::RARα* fusion gene is an extremely rare form of APL accounting for approximately 0.28% of APL patients [7] with only 18 cases having been reported in the literature [6] (Table 1). Patients with this variant typically present with morphologic and immunophenotypic features comparable to *PML::RARα* positive APL. This variant is more commonly seen in males and has a poor prognosis [7]. Due to its infrequency and the limited number of documented cases, there is little evidence for the most appropriate treatment.

**Table 1:** Summary of clinical characteristics, karyotype and breakpoint associated with all known cases of APL with a *STAT5b::RARα* fusion gene

Author and year of publication	Age	Gender	Karyotype	Breakpoint	Therapy	Course	Outcome	Effective treatment	OS (mo)
Arnould et al. 1999 [8]	67	M	45,X,-Y,add(17)(q?)[40]	Fusion event between exon 15 of *STAT5b* and exon 3 of *RARa*	ATRA	1	NR	NONE	NA
Gallagher et al. 2004 [9]	57	M	46, XY, t(10;11)(q22:q25), i(17)(q10)	Fusion event between exon 15 of *STAT5b* and exon 3 of *RARa*	DA + TR ATO + DA + TR Gemtuzumab ozogamicin ALLO-BMT	1 1 1	NR CR Relapse AT 9 MO CR Died of TRC	ATO + DA + TR Gemtuzumab ozogamicin	18 MO
Kusakabe et al. 2008 [10]	42	M	46,X,-Y, + 11[9]/46,XY [11],	Fusion event between exon 15 of *STAT5b* and exon 3 of *RARa*	ATRA + TR DA + MIT + vp16 HD-ARA-C	1 1 6	NR CR CR	DA + MIT + VP16	21 MO
Iwanga et al. 2009 [11]	41	M	47,XY,(del9),(q?),add(17) (q12), + mar1 [3].48,XY, idem,mar1[17]	Fusion event between exon 15 of *STAT5b* and exon 3 of *RARa*	ATRA + IA + TR ATRA + IA GD-Ara C + intrathecal chemotherapy + whole brain irradiation	1 2 1	CR First relapse second CR for 6 MO Second relapse: BM, died	ATRA + IA	17 MO
Cahill et al. 2011 [12]	29	F	46,XX,t(3;17)(q26;q21)	NA	ATRA + IA + TR FLA allo-BMT	1 1	NR CR Died of TRC	ATRA + IA allo-BMT	15 MO
Qiai et al. 2011 [13]	32	M	46,XY	Fusion event between exon 15 of *STAT5b* and exon 3 of *RARa*	ATRA + ATO + MIT FLAG FLAG allo-HSCT	1 1 1	NR CR CR	FLAG allo HSCT	28MO after allo HSCT ongoing
Chen et al. 2012 [14]	26	M	46, XY	Fusion event between exon 15 of *STAT5b* and exon 3 of *RARa*	ATRA IA ATRA + ATO CAG	1 1 1 1	NR NR NR NR	NONE	6 MO
Strehl et al. 2013 [15]	17	M	46,XY,t(10;11)(q22;q25),i(17) (q10)	Fusion event between *STAT5B* exon 15 and *RARa* exon 3	ATRA DA Gemtuzumab ozogamicin allo-SCT	1 1 1	NR CR, First relapse BM 9 mo Second CR	DA gemtuzumab ozogamicin	18 MO
Kluk et al. 2015 [16]	47	M	45, X, -Y/46,XY	Fusion event between *STAT5B* exon 15 and *RARa* exon 3	ATRA DA HSCT	1 1 1	NR NR CR	HSCT	9 MO
Wang et al. 2015 [17]	22	M	46,XY,inv.(9)(p13q13)c[18]/ 44–45,XY,inv.(9)(p13q13)c [cp4]/ [22	Fusion event between exon 16 of *STAT5b* and exon 3 of *RARa*	ATRA ATO + IA Refused further treatment	1 1	NR NR died	None	9 MO
Liu et al. 2016 [18]	49	M	46,XY[20]	Fusion event between exon 15 of *STAT5b* and exon 3 of *RARa*	ATRA + ATO + idarubicin IA HHT + MD-Ara-C Idarubicin + MD-Ara-C FLAG HHT + MD –Ara-C	1 4 1 1 1 1	Crm First relapse BM NR NR	ATRA + ATO + IA HHT + MD-Ara-C	> 15 mo
Zhang et al. 2018 [19]	28	F	46,XX[20	Fusion event between exon 15 of *STAT5b* and exon 3 of *RARa*	ATRA + idarubicin ATO/ATRA + DA HA + ATRA	1 1 1	NR NR DIED	NONE	2MO
Pessina et al. 2017 [20]	32	M	45,X,Y,der(17)inv(17) (p11.2q21)del(17) (q21)/46,XY	Fusion event between exon 15 of *STAT5b* and exon 4 of *RARa*	IA FLAG-IDA ALLO-HSCT	1 2	NR NR	ALLO-HSCT	12 MO AFTER HSCT:ON-GOING
Wang et al. 2018 [21]	47	M	46, XY, t(5;7)(q22;q31)/ 46,XY,der(11)/46,XY	Fusion event between exon 15 of *STAT5b* and exon 3 of *RARa*	ATRA/mitoxantrone + ATO IA; decitabine + AA/IA	1 6	NR Crm	Decitabine + AA/IA	12 MO ON-GOING
Ciangola et al. 2019 [5]	47	M	46,XY,der(5)t(5;8)	Fusion event between exon 14 of *STAT*5b and exon 3 of *RARa*	ATRA DA FLAG-IDA + ATRA Allo-HSCT	1 1 1	NR NR CR Died of multiple organ failure	FLAG-IA + ATRA	NA
Peterson et al. (2019) [22]	27	M	46, XY	Fusion event between exon 15 of *STAT5b* and exon 3 of *RARa*	ATRA + Hydroxyurea	1	DEAD	NONE	3.5 Days
Wang et al. 2020 [23]	62	M	43,-46,XY, + 2,-5, + 8,14p + ,-16,17q-,17q + , + 18,-19,-20,-21, + mar1, + mar2[CP5]/46,XY[15	NA	ATRA, ATRA + ATO ATRA + ATO + Idarubicin decitabine + AA	1 1 4 1	CR CR AT 4 MO molecular relapse at 5 mo CRm	ATRA, ATO, idarubicin, Decitabine + CAG	Ongoing at 8MO current status NA
Zhang et al. 2022 [6]	38	F	46, XX, del (6) (q22),—7,—14, + mar2 [20]	Fusion event between exon 15 of *STAT5b* and exon 3 of *RARa*	IA + CAG + ATRA + ATO AZA VEN + HA + CAG HSCT	2 2 3 1	NR NR CR	VEN + HA + CAG HSCT	6MO current status NA
Current case	61	M	*47,XY,i(17)(p10),* + *der(17)*[11]	*Fusion event between* *exon 15 of STAT5b and exon 3 of RARa*	ATRA + ATO LDAC + Ven	1 5	NR Cytogenetic remission after cycle 2 Relapse BM, died	LDAC + Ven	7 MO

*AA* aclarubicin and cytarabine, *AZA* azacitidine, *ATO* arsenic trioxide, *ATRA* all-trans retinoic acid, *CAG* cytarabine, G-CSF, and aclarubicin, *Ven* venetoclax, *LDAC* low-dose cytarabine, *CR* complete remission, *DA* daunorubicin and cytarabine, *FLAG* fludarabine, cytarabine, and recombinant human granulocyte colony stimulating factor (G-CSF), *IA* idarubicin and cytarabine, *M* male, *F* female, *MA* mitoxantrone and cytarabine, *NA* not available, *NR* no response, *OS* overall survival measured as time from date of disease diagnosis to death, *vp-16* etoposide, *CRm* molecular complete remission, *TRC* transplant-related complications

## Case report and methods

**Fig. 1:**
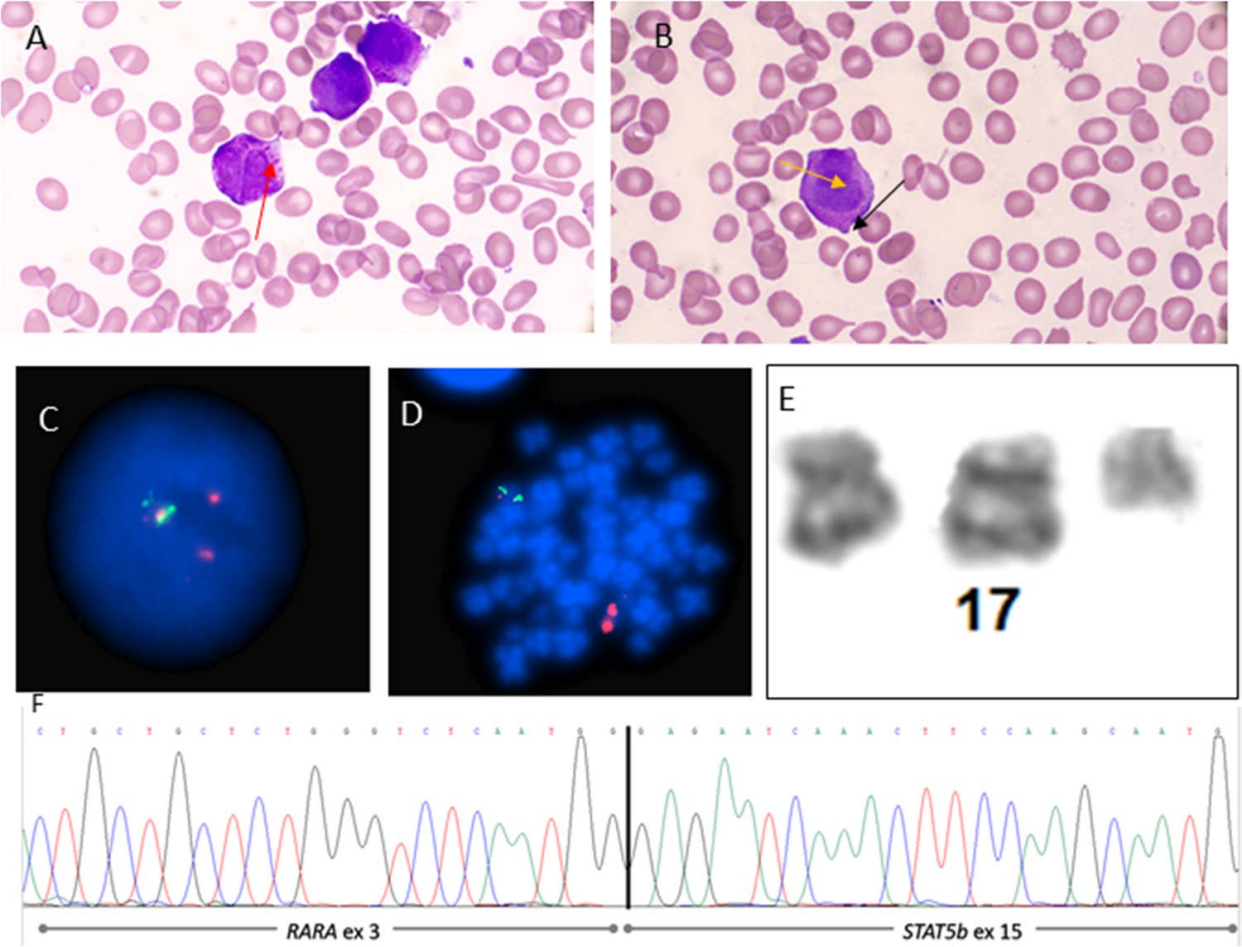
Initial bone marrow aspirate morphology and cytogenetic analysis of the patient. **A** and **B** Bone marrow aspirate showing hypercellularity and a population of medium-sized blasts with budded cytoplasm (black arrow), nuclear folding (yellow arrow), and some with Auer rods (red arrow). Pictures taken with oil at × 50 magnification. **C** Interphase nuclei showing one intact copy of RARα and two copies of the 5’ portion of the RARα probe. **D** Metaphases showing two 5’RARα signals hybridised to the der(17). **E** Isochromosome for the short arm of chromosome 17 and a derivative chromosome 17 which includes two copies of the long arms of chromosome 17. **F** Sanger sequencing of the STAT5b-RARα fusion. The Sanger sequencing at the breakpoint site is depicted by the black line

A 60-year-old male presented to the local accident and emergency department feeling generally unwell with shortness of breath, icteric sclera, and jaundice. Initial full blood count (FBC) showed pancytopenia, haemoglobin 100 g/L, white cell count 1.3 × 10^9^/L, platelet count 30 × 10^9^/L, and an absolute neutrophil count (ANC) of 0.4 × 10^9^/L. A blood film performed on peripheral blood (PB) revealed a leucoerythroblastic picture and pancytopenia. The coagulation screen and kidney function tests were normal. Serum lactate dehydrogenase (LDH) and C-reactive protein (CRP) were 492 U/L and 28 mg/L respectively. The patient had an extensive past medical history including non-ST segment elevation myocardial infarction (NSTEMI), obesity, type 2 diabetes mellitus (T2DM), hypertension, atrial fibrillation, and recurrent cellulitis. A bone marrow aspirate and trephine was performed which was grossly hypercellular, almost entirely replaced with medium/large blasts/promyelocytes with variable amounts of cytoplasm containing fine azurophilic granules, some with budded cytoplasm and many with a folded nucleus (Fig. 1A and B). These morphologic features raised suspicion of APL. Immunophenotyping analysis was performed and indicated that the blasts/promyelocytes were CD34-, HLA-DR(weak) + , MPO + , CD64 + , CD13 + ,CD33 + , and CD117 + myeloid lineage cells. As a consequence of what appeared to be classic APL morphology and immunophenotyping results, the patient was commenced on ATRA 45 mg/m^2^, awaiting urgent FISH and real-time quantitative PCR (RQ-PCR).

The patient subsequently tested negative for a *PML::RARα* rearrangement by both FISH (Fig. 1C) & RQ-PCR, following the protocol described by Europe Against Cancer (EAC). There was also no evidence of *MECOM* or *KMT2A* rearrangements. Chromosome analysis (Fig. 1E) of bone marrow cell cultures showed a karyotype consisting of 47 chromosomes including an isochromosome for the short arm of chromosome 17. There was also a derivative chromosome 17 which includes two copies of the long arms of this chromosome. ATRA treatment was discontinued at this point however subsequent FISH analysis, this time using an Vysis LSI *RARα* dual colour break-apart probe, displayed one intact copy of *RARα* along with two copies of the centromeric portion of the *RARα* probe. These copies were confirmed by metaphase FISH to be located on the der(17)—ATRA was therefore resumed pending further investigation. Three copies of *TP53* were also identified. Based on karyotyping and FISH analysis, the results therefore represented a variant *RARα* rearrangement with an unknown cryptic partner gene.

In order to identify this cryptic partner, the sample was sent for myeloid next generation sequencing (NGS). DNA libraries were prepared using the KAPA Hyper Plus Kit (Kapa Biosystems) and sequenced using a Myeloid NGS assay covering common sequence, structural and copy number variations in genes involved in myeloid neoplasms. Alignment, de-duplication, and variant calling was performed using the MyeloidTS_WF bioinformatics pipeline v1.1. This panel uncovered a cryptic inv(17) or del(17)(q21.2q21.2) *STAT5b* e15 (NM_012448.4)::*RARα* e3 (NM_000964.4) variant. The sample was also sent to Newcastle Genetics laboratory, Newcastle upon Tyne, for an RNA fusion assay using the Illumina TruSight panel and confirmed by Sanger sequencing (Fig. 1F). Therefore, based on these genomic findings, the favoured classification according to the 2022 WHO classification is “APL with a variant RARα translocation” [1]. This is also in keeping with the International Consensus Classification [24].

The patients’ disease was judged to be resistant to ATO or ATRA, and due to extensive co-morbidities, he was not a candidate for intensive chemotherapy (ICT). Therefore, he was commenced on five cycles of low dose cytarabine (LDAC), 20 mg/m^2^ given subcutaneously once daily on days 1–10 of each 28 day cycle, beginning on cycle 1 day 1, and venetoclax (Ven) 100 mg from day 4 to day 28 orally with an azole after dose escalation from days 1–4. The bone marrow biopsy following cycle 1 was hypocellular with no excess of blasts/promyelocytes, and the patient was commenced on daily granulocyte colony-stimulating factor (G-CSF). Morphological remission (MR) was achieved at this point, demonstrating a clear sensitivity to a LDAC/Ven regimen. However, *STAT5b::RARα* mutant transcripts were still detectable by molecular minimal residual disease (MRD) and flow MRD was also positive. Cycle 2 was well tolerated. However, venetoclax dosage was reduced to 14 days as a consequence of prolonged cytopenias. Cytogenetic remission was achieved following this cycle. The patient proceeded with cycles three and four respectively with a delay in cycle five due to cytopenias and probable cellulitis. A subsequent bone marrow aspirate unfortunately revealed progressive disease morphologically (approximately 33% blasts) and continued MRD positivity. The patient was transferred to ICU with neutropenic sepsis with gram-positive cocci in culture and was treated with multiple broad spectrum antibiotics. The patient continued to deteriorate with persistent pyrexia and pancytopenia requiring transfusion support and died in ICU.

## Discussion

This case highlights the complications that this variant of APL poses for both diagnosis and management. Consistent with other documented cases, this patient tested negative for a *PML::RARα* rearrangement by both FISH and RQ-PCR. Importantly, subsequent FISH testing used a *RARα* break apart probe that successfully identified the *RARα* 3’ deletion that often coincides with a *STAT5b* partner. The confirmation and precise identification of this cryptic *RARα* partner depended heavily on the custom NGS panel without which, identification of this partner would rely on the availability of a highly precise RQ-PCR assay or RNA sequencing [16]. Whilst the majority of commercially available DNA based myeloid NGS panels cannot detect structural variants and therefore will not exclude the presence of rare fusion genes such as that described in this case, the accessibility of this particular assay and its ability to detect rare structural variants, prompted specific confirmatory testing and swift application of the most suitable clinical management for this patient.

A comprehensive literature review of reported *STAT5b::RARα* positive APL cases, of which there were only 18, conducted by Zhang et al. [19] reported a median age at diagnosis of 39 ranging from 17 to 67 years of age and a greater propensity in the male population (15 patients were male and 3 were female). The majority of the treatment regimens employed across the 18 previous cases involved ICT using differing combinations of doxorubicin (DA), idarubicin (IA), fludarabine, mitoxantrone (MIT), fludarabine, cytarabine and G-CSF (FLAG), homoharringtone, and decitabine, achieving complete remission (CR) in 12 of the 18 patients. The median overall survival time was 9.5 months with a disease-free survival time of 3 months (Table 1). Whilst evidently ICT followed by HSCT is the preferred strategy utilised in patients with AML who are deemed fit, effective treatment options are, to date, extremely limited for those who are not suitable for HSCT. Our patient was ineligible for HSCT and ICT due to extensive co-morbidities.

Results from a phase 3 clinical trial conducted by Wei et al. [25] into the use of the combination LDAC/Ven in newly diagnosed AML, ineligible for ICT, demonstrated that this regime exhibits a well-tolerated safety profile and shows positive impacts on overall survival (OS), rates of remission, event free survival (EFS), transfusion requirements, and patient-reported outcomes compared with LDAC alone. This case represents, to the best of our knowledge, the first documented use of a LDAC/Ven regime in this extremely rare subtype of APL. Evidently, our patient showed a promising first-line response and sensitivity to this regime achieving MR after cycle 1. Further understanding of the mechanisms behind the anti-leukaemic activity and demonstrated disease sensitivity to a combined LDAC/Ven regime should be explored in an attempt to define a precise empirical treatment for this extremely rare subtype of APL, particularly in ineligible ICT candidates.

Although clearly effective for this patient, the use of LDAC/Ven is not the only therapy suitable for high-risk AML patients. Given the CD33 positivity of the present case the use of gemtuzumab ozogamicin (GO) may have been a potential option. GO is an anti-CD33 antibody conjugated to calicheamicin that has shown to be effective for the treatment of high-risk APL in recent clinical trials [26]. However, patients on this trial received ICT post-remission with a third of the recruited patients unable to complete prescribed regimens [3]. Unfortunately, this patient was not able to tolerate increased dosages or duration of therapy and deteriorated quickly before additional treatment strategies could be employed.
